# MFG-E8 Alleviates Cognitive Impairments Induced by Chronic Cerebral Hypoperfusion by Phagocytosing Myelin Debris and Promoting Remyelination

**DOI:** 10.1007/s12264-023-01147-1

**Published:** 2023-11-18

**Authors:** Xiaohong Dong, Zhi Zhang, Xin Shu, Zi Zhuang, Pinyi Liu, Renyuan Liu, Shengnan Xia, Xinyu Bao, Yun Xu, Yan Chen

**Affiliations:** 1 Department of Neurology, Nanjing Drum Tower Hospital, Affiliated Hospital of Medical School, Nanjing University, Nanjing, 210008 China; 2 Department of Neurology, Nanjing Drum Tower Hospital Clinical College of Traditional Chinese and Western Medicine, Nanjing University of Chinese Medicine, Nanjing, 210008 China; 3 Department of Neurology, Drum Tower Hospital of Nanjing Medical University, Nanjing, 210008 China; 4 Jiangsu Key Laboratory for Molecular Medicine, Medical School of Nanjing University, Nanjing, 210008 China; 5 Jiangsu Provincial Key Discipline of Neurology, Nanjing, 210008 China

**Keywords:** White matter injury, Cognitive dysfunction, MFG-E8, Remyelination, Microglial phagocytosis

## Abstract

Chronic cerebral hypoperfusion is one of the pathophysiological mechanisms contributing to cognitive decline by causing white matter injury. Microglia phagocytosing myelin debris in a timely manner can promote remyelination and contribute to the repair of white matter. However, milk fat globule-epidermal growth factor-factor 8 (MFG-E8), a microglial phagocytosis-related protein, has not been well studied in hypoperfusion-related cognitive dysfunction. We found that the expression of MFG-E8 was significantly decreased in the brain of mice after bilateral carotid artery stenosis (BCAS). MFG-E8 knockout mice demonstrated more severe BCAS-induced cognitive impairments in the behavioral tests. In addition, we discovered that the deletion of MFG-E8 aggravated white matter damage and the destruction of myelin microstructure through fluorescent staining and electron microscopy. Meanwhile, MFG-E8 overexpression by AAV improved white matter injury and increased the number of mature oligodendrocytes after BCAS. Moreover, *in vitro* and *in vivo* experiments showed that MFG-E8 could enhance the phagocytic function of microglia via the α_V_β_3_/α_V_β_5_/Rac1 pathway and IGF-1 production to promote the differentiation of oligodendrocyte progenitor cells into mature oligodendrocytes. Interestingly, we found that MFG-E8 was mainly derived from astrocytes, not microglia. Our findings suggest that MFG-E8 is a potential therapeutic target for cognitive impairments following cerebral hypoperfusion.

**Supplementary Information:**

The online version contains supplementary material available at 10.1007/s12264-023-01147-1.

## Introduction

Chronic cerebral hypoperfusion (CCH) is one of the pathophysiological mechanisms resulting in cognitive impairment and degenerative processes contributing to dementia [1]. The severity of hypoperfusion correlates with the degree of dementia and also predicts the conversion from mild cognitive impairment to dementia [2, 3]. White matter injury (WMI) induced by CCH is the main pathogenic mechanism of cognitive impairment [4, 5]. White matter hyperintensities are frequently detected in patients with cerebral hypoperfusion, including vascular cognitive impairment, cerebral small vessel disease, and severe carotid artery stenosis [5–7]. Moreover, the burden of white matter hyperintensities predicts cognitive decline, especially impairment of attention and executive functions [8]. Thus, we proposed that repairing WMI could provide vital opportunities to prevent the cognitive decline caused by CCH.

The histopathology of ischemic WMI is demyelination attributed to cerebral hypoperfusion [9, 10]. Remyelination is a self-repair process for WMI, in which the insulating myelin sheath is restored to axons, thereby facilitating recovery from functional loss. Remyelination relies on oligodendrocyte precursor cells (OPCs) differentiating into oligodendrocytes (OLs) to synthesize the new myelin sheath, and this is hindered by myelin debris from demyelination [11]. The removal of myelin debris by microglia, CNS resident immune cells, promotes remyelination and improves cognitive impairment [12].

Milk fat globule-epidermal growth factor-factor VIII (MFG-E8), a ligand for the integrins α_V_β_3_ and α_V_β_5_, acts as an intermediary factor that mediates the recognition and clearance of phagocytes by microglia [13]. In the focal brain ischemia model, MGF-E8 mediates microglial phagocytosis of neurons exposed to phosphatidylserine [14]. MFG-E8 also mediates the phagocytosis of Aβ by microglia in primary glial cell culture, which shows the potential treatment value of MFG-E8 in Alzheimer’s disease [15]. However, whether MFG-E8 can promote the phagocytosis of myelin debris by microglia and thus promote remyelination remains unclear.

Here, we used the BCAS model to stimulate WMI after chronic cerebral hypoperfusion. We showed that MFG-E8 knockout reduced myelin debris clearance by microglia *in vivo*, exacerbated WMI, and worsened cognitive impairment. These events resulted from decreased OPC recruitment and differentiation into mature OLs, eventually inhibiting remyelination. This provides the proof of concept that MFG-E8 could promote remyelination, suggesting this as a potential novel therapeutic avenue in cognitive impairment after CCH.

## Materials and Methods

### Mice

MFG-E8^−/−^ mice (C57BL/6 background), purchased from the Model Animal Research Center of Nanjing University, were mated from heterozygotes, and littermate MFG-E8^+/+^ mice were used as controls. Mice were housed in specific pathogen-free animal facilities at the Affiliated Drum Tower Hospital of Nanjing University Medical School. All animal experiments were performed in accordance with institutional guidelines and approved by the Animal Care Committee of Nanjing University.

### Microglia and Astrocyte Culture

Primary microglia were obtained as previously described [16]. Briefly, C57BL/6J neonatal pups (P0–P1) were used. After removing meninges and visible blood vessels, the brains were digested with 0.25% trypsin in a 37 °C incubator for 10 min and then the same amount of Dulbecco’s modified Eagle’s medium (DMEM) supplemented with 10% fetal bovine serum and 100 μg/mL streptomycin was used to terminate digestion. The cell suspension was seeded in 75 cm^2^ flasks and maintained at 37 ℃ in a humidified incubator with 5% CO_2_. The culture medium was changed every three days. After 10 days–12 days, loosely attached microglia were harvested from the medium by shaking the flasks for 10 min. After two microglia harvests, astrocytes were digested by trypsin and prepared for follow-up experiments.

### OPC Culture

The OPCs were prepared from the cerebral cortices of newborn C57BL/6 pups. Briefly, after removing meninges, cortexes were dissected and then dissociated by mechanical trituration. Later, the suspension was filtered through a 70 μm nylon cell strainer and plated on plates coated with poly-D-lysine (Sigma, USA). The cells were cultured in a proliferation medium (DMEM/F12 containing B27, penicillin/streptomycin, 5 ng/mL basic fibroblast growth factor, and 15 ng/mL platelet-derived growth factor-AA). After 7 days–10 days, the medium was changed to a differentiation medium containing T3 (triiodothyronine, R&D systems, USA) and ciliary neurotrophic factor (GenScript) to induce the differentiation of OPCs. Conditioning medium from microglia was added into primary OPCs for further experiments.

### Primary Neuron Culture

The primary neuron culture was prepared from E16 C57/BL6J mouse embryos as previously reported [17]. The cells were cultured in a Neurobasal medium with 1X B27 for 8 days–10 days. Then the mRNA of the neurons was extracted for the next experiments.

### BCAS

C57BL/6 or MFG-E8 knockout mice weighing 25 g–28 g were used for BCAS surgery. The mice were anesthetized with 4% chloral hydrate (10 mL/kg) by intraperitoneal injection. The BCAS model was established as previously described [18]. Briefly, after exposing both common carotid arteries (CCAs) from their sheaths, a microcoil with a 0.18-mm inner diameter (Sawane Spring Co) was twined by rotating it around the right CCA. After 30 min, another micro-coil of the same size was twined around the left CCA.

### Stereotaxic Intracranial Injection

A stereotaxic intracranial injection was performed following established protocols [19]. The injection site for the corpus callosum (CC) was determined based on the brain map, with coordinates of 1 mm lateral from the midline, 0.8 mm anterior from the bregma, and 2.2 mm deep from the bregma. To initiate the procedure, mice were anesthetized using 2.5% avertin. A small hole was drilled in the skull, and subsequently, 400 nL (200 nL per hemisphere) of MFG-E8-overexpressing AAVs (pAAV-CMV-Mfge8-3xFlag-P2A-mNeonGreen-tWPA, OBiO, China) or control AAVs (pAAV-CMV-3xFlag-P2A-mNeonGreen-tWPA, OBiO, China) were bilaterally injected into the CC. To prevent viral leakage, the micropipette was kept in place for 5 min after injection. Once the wound was sutured, the mice were allowed to regain consciousness on a thermostatic heat source.

### Novel Object Recognition

The working memory was evaluated in each group of mice by using new object recognition (NOR). During habituation, the mice were allowed to explore an empty box. Three days after habituation, they were presented with two similar objects during the first session. Then one of the two objects was replaced by a new object during a second session. The time taken to explore the new object provided an index of recognition memory.

### Open Field Test

The open field test (OFT) was used to measure general locomotor activity and anxiety-like behavior. Each mouse was gently placed into a corner of the open field box (40 cm × 40 cm × 40 cm) to explore freely for 10 min. The test sessions were recorded by a video camera installed on the ceiling above the apparatus. The total distance moved, mean velocity and corner time were recorded.

### Fear Conditioning Test

The fear conditioning test was applied as described previously. The mice were placed in a conditioning chamber (Panlab, Spain) and allowed to freely explore it for 5 min. Then a 30-s tone (80 dB) was delivered followed by a 2-s foot shock (0.75 mA). After that, the mouse stayed in the chamber for another 1 min to evaluate post-shock freezing. Context-dependent memory was evaluated on the next day. The mice were again placed in the same chamber, but without any stimulation, and scored for the freezing behavior. The cue-dependent memory was examined in a novel chamber. The mice were placed in a novel chamber to freely explore for 1 min without any stimulation followed by 4-min tone-stimuli. Freezing was defined as a completely immobile posture except for respiration, and the freezing time was determined using Packwin software (Panlab, Spain).

### MRI

Mice before and after BCAS surgery received an MRI scan on a 9.4T Bruker MR system (BioSpec 94/20 USR, Bruker) using a 440-mT/m gradient set, an 86-mm volume transit RF coil, and a single channel surface head coil. The mice were anesthetized by inhalation of 3% isoflurane before scanning. The T2-weighted imaging consisted of the following sequence: repetition time (TR) = 2500 ms, echo time (TE) = 33 ms, field of view (FOV)= 2 × 2 cm, matrix = 256 × 256, and slice thickness = 0.7 mm. diffusion tensor imaging was applied using the following spin-echo echo-planar imaging sequence: Two b-values (b = 0 and 1000 s/mm^2^) along with 30 non-collinear directions, δ = 4.1 ms, Δ = 10.3 ms; TR = 1500 ms, TE = 23.27 ms, FOV = 20 mm × 20 mm, matrix = 128 × 128, and 22 adjacent slices of 0.7 mm slice thickness. MRIcron was used to process the imaging data. Diffusion data were post-processed using the FSL (v.5.0.9) pipeline, consisting of corrections for eddy currents and movement artifacts (eddy_correct), rotations of gradient directions according to eddy current corrections (fdt_rotate_bvecs), brain mask extractions based on b0 images (bet), and fractional anisotropy (FA) map calculations by fitting a diffusion tensor model at each voxel (drift). CC areas were drawn using ITK-SNAP to extract the FA values. The heatmap was generated at the Institute of High Energy Physics, Chinese Academy of Sciences.

### Electron Microscopy

Mice were subjected to transcardial perfusion with phosphate-buffered saline (PBS) and 4% phosphate-buffered paraformaldehyde (PFA). The CC samples were incubated overnight in 2.5% glutaraldehyde. Then the brains were postfixed in a mixture of 1% osmic acid and 1.5% potassium ferricyanide for 1 h at 4 ℃. After alcohol gradient dehydration, the samples were embedded in epoxy resin, and stained with uranyl acetate and lead citrate. Images were acquired using transmission electron microscopy (Hitachi, HT7800). The g-ratio was calculated by dividing the axonal diameter by the myelinated fiber diameter. Thirty myelinated axons were randomly analyzed across multiple fields per mouse to calculate the g-ratio.

### Immunofluorescence Staining and Imaging

Mice were anesthetized and transcardially perfused with cold PBS and cold 4% PFA. The dissected brains were post-fixed in 4% PFA for 24 h at 4 ℃ and subsequently cryoprotected in 30% sucrose for 72 h at 4℃. Frozen sections were cut at 20 μm for use. Cells were fixed in 4% PFA for 30 min and washed three times with PBS. Sections or cells were blocked with 5% BSA and 0.25% Triton X-100 and incubated overnight at 4 ℃ with primary antibodies. The primary antibodies were as follows: Oligo2 (Millipore), CC1 (Abcam), PDGFRα38 (BD Biosciences), MFG-E8 (Santa Cruz), Iba-1 (Abcam), GFAP (Proteintech), Map2 (Bioworlde), CD68 (Abcam), and degraded myelin basic protein (dMBP; Millipore). The next day, sections or cells were incubated with appropriate secondary antibodies at room temperature for 2 h. Fluorescence images were obtained using a fluorescence microscope (Olympus IX73) or a confocal laser-scanning microscope (Olympus FV3000).

### Black-Gold Staining

Black-gold staining was performed using the Black-Gold II myelin staining kit (Biosensis, USA). Briefly, 0.3% Black-Gold II solution was prepared and preheated to 65 ℃. Brain sections were incubated with Black-Gold II solution for 10 min at 65 ℃. During staining, the slides were monitored at 2 min–3 min intervals under the microscope to stain the finest myelinated fibers. After further washing in distilled water, the sections were fixed in 1% sodium thiosulfate for 3 min at 65 ℃. After 3 washes, the sections were dehydrated in a series of graded ethanols, cleared in xylene, and cover-slipped.

### RT-PCR

Total RNA was extracted from fresh brain tissue using TRIzol (Invitrogen, USA) and was reverse-transcribed into cDNA using a PrimeScript RT reagent kit (Takara, China). Quantitative real-time PCR was performed on an ABI 7500 PCR instrument (Applied Biosystems) with a SYBR green kit (Takara, China). Relative gene expression was analyzed by 2^−(ΔΔCt)^ with normalization to GAPDH. The primer sequences were as follows:

MFG-E8 Forward: AGATGCGGGTATCAGGTGTGA

MFG-E8 Reverse: GGGGCTCAGAACATCCGTG

GAPDH Forward: AGGTCGGTGTGAACGGATTTG

GAPDH Reverse: TGTAGACCATGTAGTTGAGGTCA

### Western Blotting

Equal quantities of protein extracts were subjected to SDS-PAGE and transferred to polyvinylidene difluoride membranes (Millipore, USA). After blocking in 5% non-fat milk for 1 h at room temperature, the membranes were incubated overnight at 4°C with the following primary antibodies: MFG-E8 (Santa Cruze), NG2 (Millipore), and PLP1 (Abclone). The membranes were subsequently incubated with corresponding secondary antibodies and visualized with chemiluminescence reagents provided with an ECL kit (Bioworld, USA).

### Myelin Debris Production

Mouse myelin was prepared as previously described [18]. Whole brains were removed from 6-week-old C57Bl/6 mice and myelin was isolated using a discontinuous sucrose gradient. Brains were isolated in 0.32 mol/L sucrose solution and cut into pieces. The homogenized brain solution was gently added to the surface of 0.83 mol/L sucrose solution. After centrifuging at 100,000 r/min for 45 min at 4 °C, the interface was collected and dissolved in 35 mL Tris.Cl buffer solution, and centrifuged at 100,000 r/min for 45 min at 4 °C. The supernatant was aspirated, and the pellet re-suspended in 10 mL–15 mL of Tris.Cl buffer and then centrifuged at 100,000 r/min for 45 min. The pellet was resuspended in 5 mL–6 mL of sterile PBS and centrifuged at 22,000 r/min for 10 min at 4 °C. Finally, pure myelin debris was achieved. The purified myelin pellet was sub-packaged at a concentration of 20 mg/mL with PBS and stored at − 80 °C.

### Phagocytosis Assays

Wild-type primary microglia were plated in a 24-well plate at a density of 50,000 cells per well. The cells were incubated for 24 h at 37 °C under 5% CO_2_, and then recombinant murine MFG-E8 (200 ng/mL, R&D systems) and pHrodo-labeled myelin (100 μg/mL) were added to the medium at the same time. Then, primary microglia were digested by trypsin for flow cytometry.

### Fluorescence-activated Cell Sorting (FACS) of Microglia and Astrocytes

Mice were anesthetized and transcardially perfused with ice-cold PBS. The brains were then dissected, removing the olfactory bulb, cerebellum, and brainstem. The remaining brain tissue was cut into small pieces, digested with trypsin for 8 min, and the resulting precipitate was collected. After resuspension with debris removal solution, Hanks’ balanced salt solution was slowly added, followed by centrifugation at 3,000 g at 4 °C. The top two layers, containing myelin sheath and liquid, were discarded, and the bottom layer was transferred. This layer was washed three times with PBS, and the cells were incubated with specific antibodies (GLAST-1-PE, CD11b-APC, and CD45-PE/C7) in the dark for 30 min. The cells were then resuspended for testing. Flow cytometry analysis and cell sorting were applied using a FACSAria II (BD Biosciences). The sorted cell populations were defined as follows: other cells (CD45^-^ GLAST-1^−^), microglia (CD11b^+^CD45^median^), and astrocytes (GLAST-1^+^/CD45^−^). RNA isolation was carried out immediately after sorting using an RNA isolation kit. Post-sorting population analyses and graphical representations were generated using FlowJo software v.10 (TreeStar Inc.).

### Statistical Analysis

GraphPad Prism software version 20.0 (GraphPad Software, La Jolla, CA) was used for statistical analysis. The data are expressed as the mean ± standard deviation (SD). For independent two-group comparison, the student’s *t*-test or the Mann–Whitney U test was applied, while for paired two groups, the paired *t*-test was used. For multiple comparisons, the data were analyzed by one-way analysis of variance (ANOVA) followed by Bonferroni’s *post hoc* test. *P* < 0.05 was considered statistically significant.

## Results

### MFG-E8 Knockout Worsens Cognitive Dysfunction

**Fig. 1:**
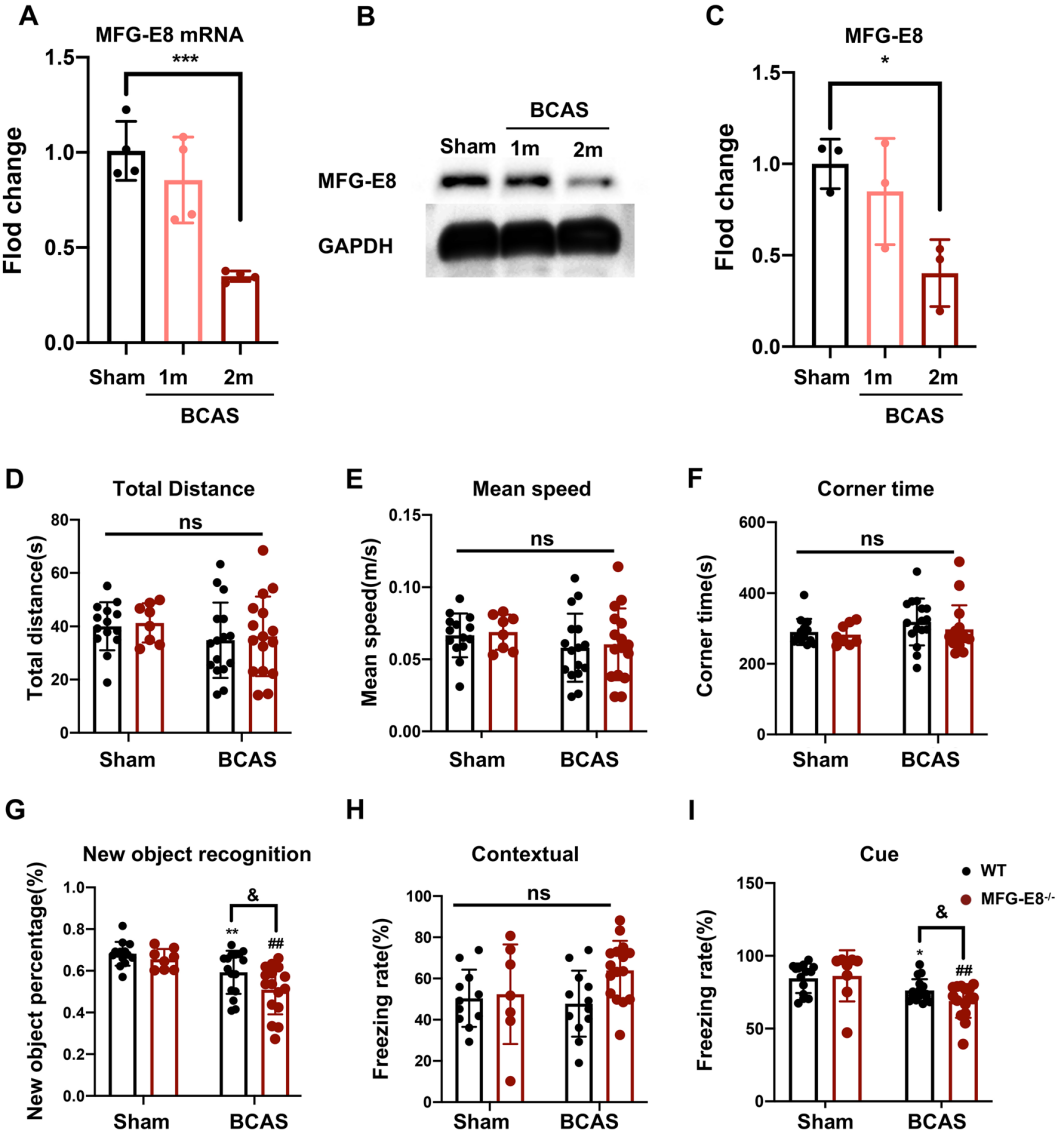
MFG-E8 expression decreases after BCAS and MFG-E8 deficiency worsens the BCAS-induced cognitive impairment. **A** Quantitative RT-PCR analysis of MFG-E8 mRNA from sham, BCAS 1 month, and BCAS 2 months mice (*n* = 4 per group, ANOVA) ***P < 0.001. **B** Representative Western blots of MFG-E8 expression. **C** Densitometry analyses of MFG-E8 expression normalized to GAPDH (*n* = 3, ANOVA, *P < 0.05). **D**–**F** The total distance, mean speed, and corner time in the open field test (ns, no significant difference). G The exploratory preference for novel objects in the new object recognition test (***P < 0.001 *vs* WT sham group, ****P < 0.0001 *vs* WT sham group, ^#^*P* < 0.05 *vs* WT BCAS group. **H**, **I** The freezing rate in the contextual cued fear conditioning test (**H**) and the cued contextual fear conditioning test (**I**) (*n* = 8–16 mice, unpaired *t*-test; *P < 0.05 *vs* WT sham group, ***P < 0.001 *vs* WT sham group, ^##^*P* < 0.01 vs MFG-E8 ^−^/^−^ sham, ^&^*P* < 0.05 vs WT BCAS group. All data represent the mean ± SD.

First of all, we tested the expression changes of MFG-E8 in cerebral white matter after BCAS, and the mRNA and protein levels of MFG-E8 in the corpus callosum were assessed in the sham group, 1 month and 2 months after the operation. The mRNA and protein expression of MFG-E8 both decreased significantly at 1 month and 2 months after BCAS, and the decrease was significant at 2 months after surgery (Fig. 1A–C).

Then, to determine the effect of reduced MFG-E8 expression on cognitive function after chronic cerebral ischemia, several behavior tests were performed in wild-type (WT) and MFG-E8^−/−^ mice. First, we conducted an open field test to exclude the influence of locomotor activity and anxiety-like behavior on cognitive function. The total distance moved and mean velocity did not differ in WT and MFG-E8^−/−^ mice both before and after BCAS surgery, suggesting that the two groups of mice had the same exploration capacity (Fig. 1D, E). Moreover, there was also no difference in the corner time, an indicator of anxiety behavior, between the two groups (Fig. 1F). Next, a new object recognition test was applied to examine the working memory. The BCAS group showed a clear decrease in discriminative ability in the test, while MFG-E8^−/−^ mice exhibited significant exacerbation in the discrimination index (Fig. 1G). The contextual and tone-cued fear conditioning tests were used to evaluate hippocampal-dependent memory and amygdala-dependent memory, respectively. In the contextual conditioning test, compared with the state before and after BCAS surgery, there was no significant difference in the freezing time of WT and MFG-E8^−/−^ mice (Fig. 1H). In the tone-cued fear conditioning test, the freezing time of MFG-E8^−/−^ mice was shorter than that of WT mice (Fig. 1I).

These results suggested that the MFG-E8 plays a protective role in cognitive repairment, especially in working memory.

### MFG-E8 Reduces BCAS-induced White Matter Damage

White matter damage is an important pathological mechanism of cognitive dysfunction after BCAS. To explore the association between the improvement effect of MFG-E8 on cognitive impairment and the repair of WMI, we used neuroimaging, Gold-Black staining, and electron microscopy to determine the degree of WMI of WT and MFG-E8^−/−^ mice.

**Fig. 2:**
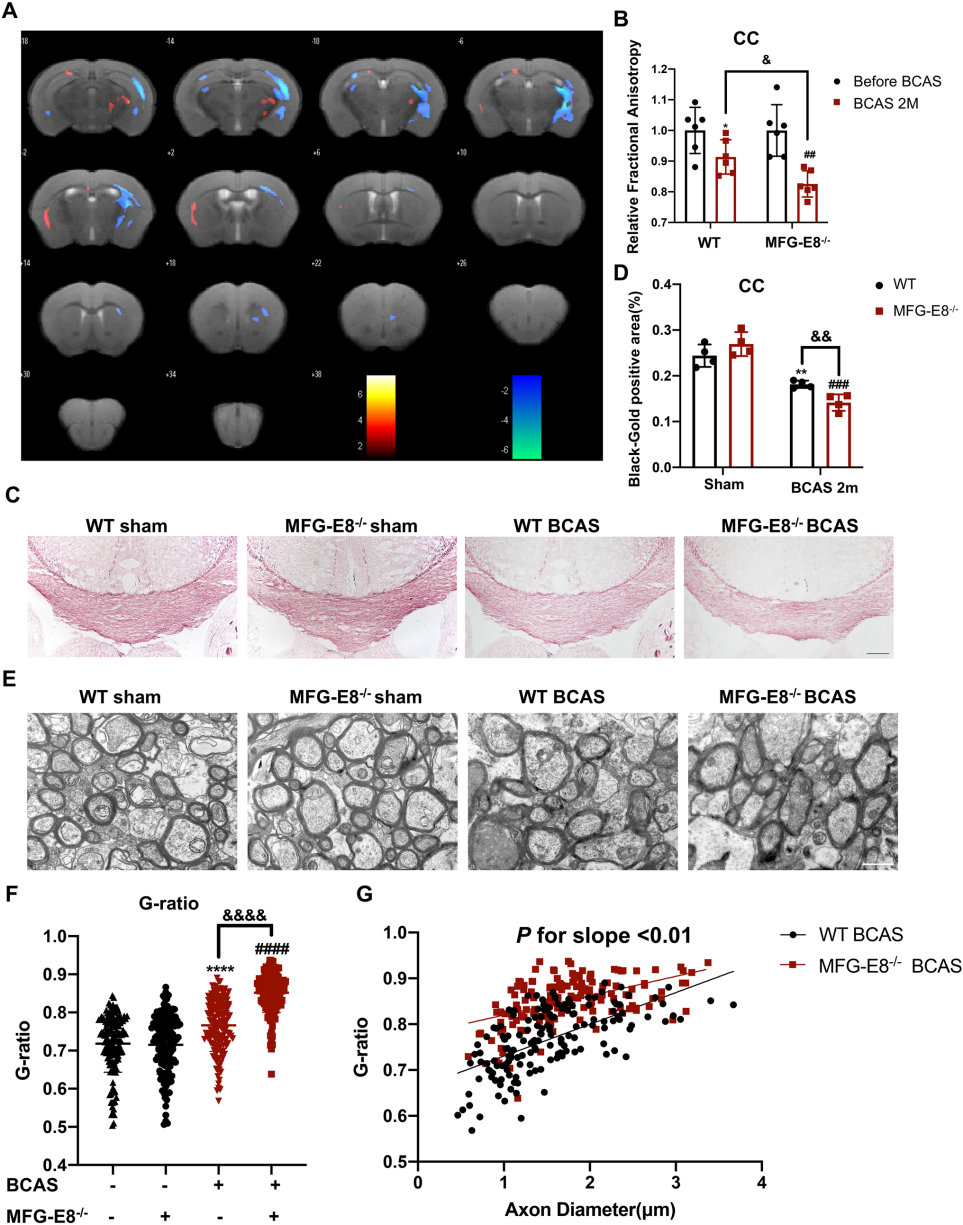
MFG-E8 knockout exacerbates white matter damage after BCAS. **A** Heatmaps generated from DTI axial views of FA acquired from WT and MFG-E8^−/−^ mice at 2 months after surgery. **B** Quantification of FA values in the corpus callosum in the WT sham, WT BCAS, MFG-E8^−/−^ sham, and MFG-E8^−/−^ BCAS groups at 2 months after surgery. The values were normalized to the mean value of the sham group (*n* = 6 per group; *P < 0.05 *vs* WT sham, paired *t* test; ^##^*P* < 0.01 *vs* MFG-E8^−/−^ sham, paired *t* test; ^&^*P* < 0.05 *vs* WT BCAS, unpaired *t* test). **C** Representative images of black-gold staining (lavender) in the corpus callosum at day 60 after BCAS. Scale bar: 200 μm. **D** Quantification of the immunofluorescent intensity of black-gold staining area in the CC (*n* = 4, unpaired* t*-test; **P < 0.01 *vs* WT sham, ^###^*P* < 0.001 *vs* MFG-E8^−/−^ sham, ^&&^*P* < 0.01 *vs* WT BCAS). **E** Representative electron microscopy images in the CC. Scale bar: 1 μm. **F** Quantification of G-ratios (*n* = 153 for WT sham mice, *n* = 144 for MFG-E8^−/−^ sham mice, *n* = 154 for WT BCAS mice, *n* = 156 for MFG-E8^−/−^ BCAS mice; ****P < 0.0001 *vs* WT sham, ^####^*P* < 0.0001 *vs* MFG-E8^−/−^ sham, ^&&&&^*P* < 0.0001 *vs* WT BCAS, unpaired *t*-test). **G** Scatterplots of the myelin g-ratio as a function of axon diameter in the corpus callosum in the WT and MFG-E8^−/−^ BCAS groups at 2 months after surgery. Axons were measured from 4 mice per group (*n* = 154 for WT BCAS mice, *n* = 156 for MFG-E8^−/−^ BCAS mice, Mann–Whitney U-test). All data are presented as the mean ± SD.

The diffusion tensor imaging (DTI) sequence is sensitive to white matter microstructural integrity by measuring the microscopic diffusion of water molecules [20]. The WT and MFG-E8^−/−^ mice were subjected to a whole-brain MRI scan including the DTI sequence before the operation and 60 days afterward. The fractional anisotropy (FA) values of both groups decreased after BCAS. Moreover, MFG-E8^−/−^ mice exhibited lower FA values than the WT group at 60 days after BCAS (Fig. 2A, B). Besides, we found that MFG-E8-deficient mice had a lower Black-Gold staining intensity in the CC area than WT mice at 60 days after BCAS (Fig. 2C, D). Furthermore, to compare the ultrastructure of the myelin between the two groups, electron microscopy was used. G-ratio analysis revealed significantly decreased myelin sheath thickness in MFG-E8^−/−^ mice compared to WT mice 2 months after BCAS (Fig. 2E–G).

The above data suggested that MFG-E8 can repair WMI after chronic cerebral hypoperfusion.

### MFG-E8 Deficiency Reduces the Number of Myelin-producing Oligodendrocytes

**Fig. 3:**
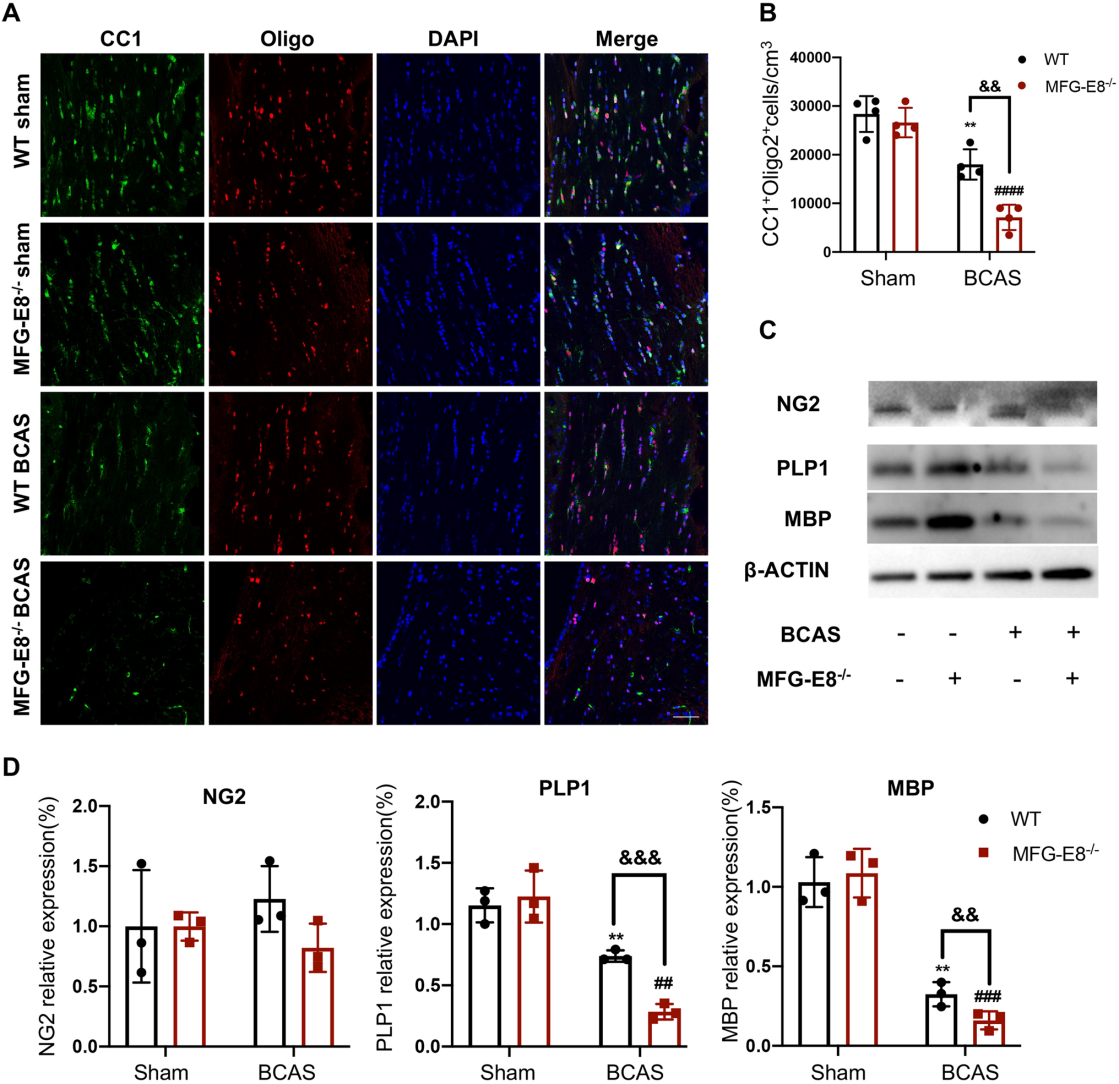
MFG-E8 deficiency hinders OPCs from differentiating into mature oligodendrocytes. **A** Representative immunostaining of CC1 and Olig2 in the corpus callosum in the WT sham, MFG-E8^−/−^ sham, WT BCAS at 2 months, and MFG-E8^−/−^ BCAS at 2 months after surgery. Nuclei are stained with DAPI. Scale bar: 50 μm. **B** The density of CC1^+^Olig2^+^ cells in the corpus callosum in the above four groups (*n* = 4 per group **P < 0.01 *vs* WT sham, ^####^*P* < 0.0001 *vs* MFG-E8^−/−^ sham, ^&&^*P* < 0.01 *vs* WT BCAS, unpaired *t*-test). **C** Western blots showing the expression of NG2, PLP1, and MBP in the corpus callosum with GAPDH as a loading control. **D** Densitometry analyses of NG2, PLP1, and MBP expression normalized to GAPDH (*n* = 3 per group; **P < 0.01 *vs* WT sham, ^##^*P* < 0.01, ^###^*P* < 0.001 *vs* MFG-E8^−/−^ sham, ^&&^*P* < 0.01, ^&&&^*P* < 0.001 *vs* WT BCAS, unpaired *t*-test. All data are presented as the mean ± SD).

OLs and wrapped axons are the main components of cerebral white matter. OL injury can be followed by a robust regenerative response, which gives rise to the formation of new myelin sheaths—a process termed remyelination. Remyelination depends on OPCs differentiating and maturing into myelinating OLs. To explore whether MFG-E8 promotes remyelination after chronic cerebral ischemia, we applied immunofluorescence staining for Olig2, an OL lineage cell marker, and CC1, a mature OL marker. The numbers of CC1^+^Oligo2^+^ OLs in the corpus callosum were decreased after BCAS compared to the sham group. Moreover, MFG-E8-deficient mice showed fewer of those mature OLs (Fig. 3A, B).

Furthermore, we measured the protein expression of different OL lineage markers in the corpus callosum of WT and MFG-E8^−/−^ mice. The expression of NG2, a marker for OPCs, did not significantly differ between WT and MFG-E8^−/−^ mice at 60 days after surgery. However, the expression of mature OL markers (MBP and PLP1) showed a significant decline in the MFG-E8^−/−^ mice compared to WT mice at 60 days after BCAS (Fig. 3C, D). These results indicated that MFG-E8 enhances the numbers of mature OLs and plays a promotion role in remyelination after BCAS.

### MFG-E8 Overexpression Reduces White Matter Damage and Enhances Oligodendrocytes Maturation

**Fig. 4:**
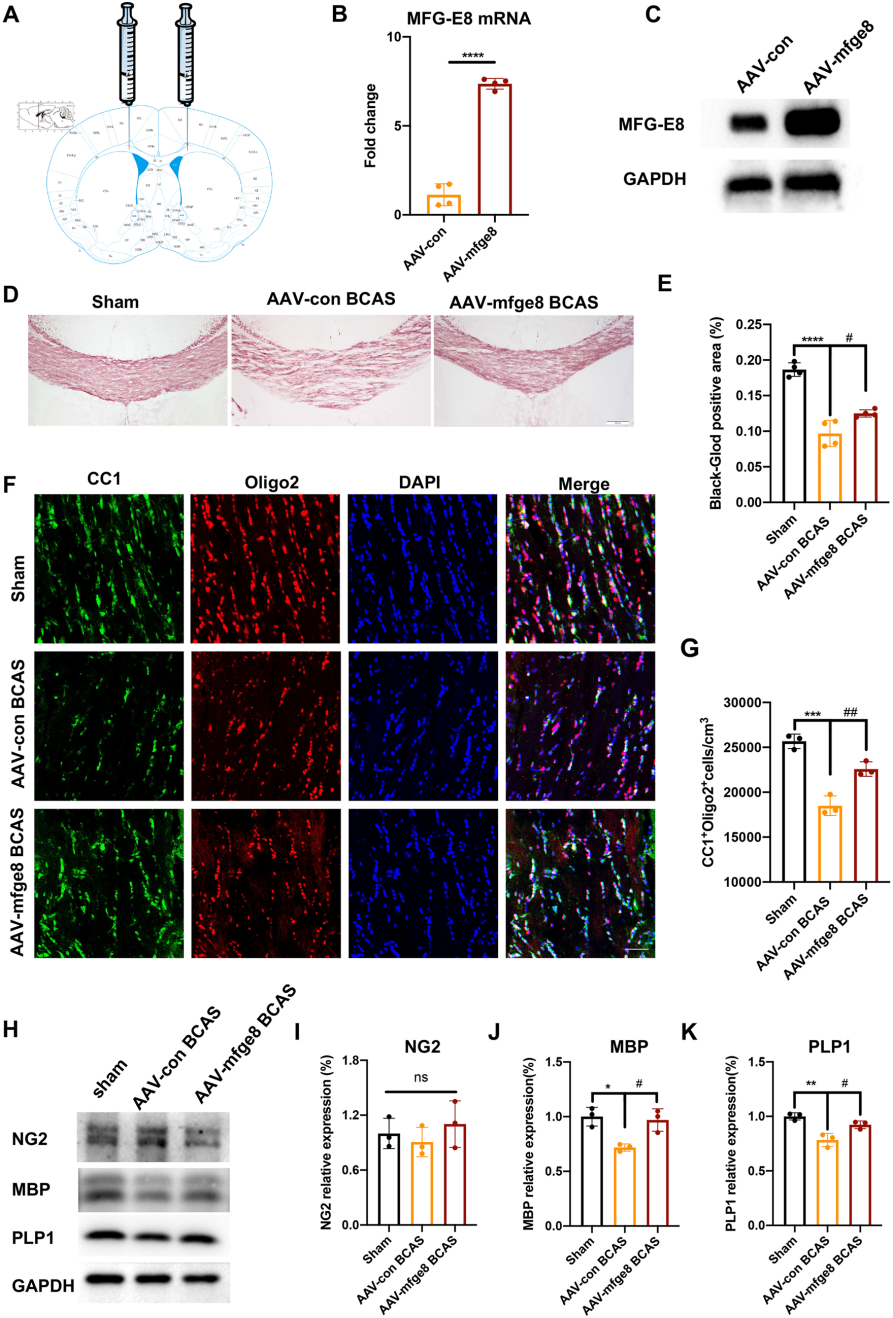
Overexpression of MFG-E8 alleviates white matter damage and promotes oligodendrocyte maturation. **A** Diagram of pAAV-CMV-Mfge8-3xFlag-P2A-mNeonGreen-tWPA injection into the CC. **B** PCR analysis of MFG-E8 expression in the CC at 3 weeks after stereotaxic intracranial injection (*n* = 4 per group; ****P < 0.0001, unpaired *t*-test). **C** Representative Western blots of MFG-E8 expression in the CC at 3 weeks after stereotaxic intracranial injection. **D** Representative images of black-gold staining (lavender) in the CC at day 60 after BCAS. Scale bar: 200 μm. **E** Immunofluorescent intensity of the black-gold staining area in the CC (*n* = 4, ANOVA; ****P < 0.0001 *vs* WT sham, ^#^*P* < 0.05 *vs* AAV-con BCAS. **F** Representative immunostaining of CC1 and Olig2 in the CC in the WT sham, AAV-con BCAS at 2 months, and AAV-mfge8 BCAS at 2 months after surgery. Nuclei are stained with DAPI. Scale bar: 50 μm. **G** Density of CC1^+^Olig2^+^ cells in the CC in the above three groups (*n* = 3 per group; ***P < 0.001 *vs* WT sham, ^##^*P* < 0.01 *vs* AAV-con BCAS, ANOVA). **H** Western blots showing the expression of NG2, MBP, and PLP1 in the CC with GAPDH as a loading control. **D** Densitometry analyses of NG2, MBP, and PLP1 expression normalized to GAPDH (*n* = 3 per group; *P < 0.05, **P < 0.01 *vs* WT sham, ^#^*P* < 0.05 *vs* AAV-con BCAS, ANOVA. All data are presented as the mean ± SD).

Given the decrease of MFG-E8 expression after BCAS, we overexpressed MFG-E8 by stereotactically injecting AAV into the CC to verify the positive role of MFG-E8 in WMI and remyelination (Fig. 4A). The overexpression of MFG-E8 was confirmed by Q-PCR and Western Blotting (Fig. 4B, C). We found that mice overexpressing MFG-E8 had less white matter damage induced by BCAS through Black-Gold staining when compared to mice injected with the control virus (Fig. 4D, E). Furthermore, immunofluorescence analysis revealed an increase in mature OLs (CC1^+^Oligo2^+^) in MFG-E8-overexpressing mice after BCAS compared to AAV-Con mice (Fig. 4F, G). In addition, Western blot results demonstrated a significant upregulation of mature OL markers, such as MBP and PLP1, in MFG-E8-overexpressing mice after BCAS, compared to control mice (Fig. 4H, K). These results further showed that MFG-E8 can improve white matter damage.

### MFG-E8 Enhances Myelin Debris Removal by Activating Microglial α_V_β_3_ and α_V_β_5_ Receptors

**Fig. 5:**
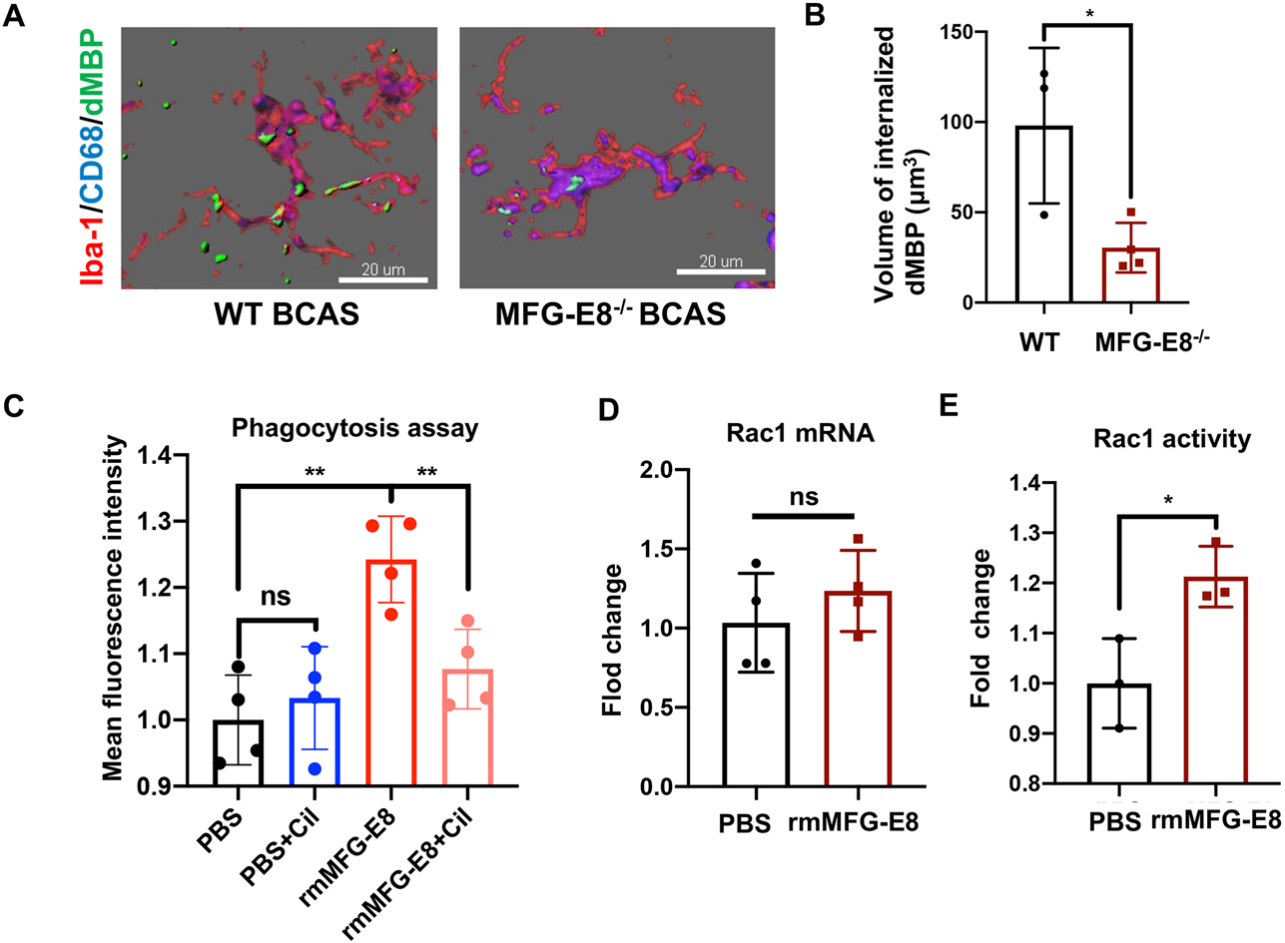
MFG-E8 enhances the phagocytosis of myelin debris by binding to microglial α_V_β_3_ and α_V_β_5_ receptors. **A** 3D-confocal images of CD68^+^(blue) Iba-1^+^(red) microglia phagocytosing MBP debris (green) in WT and MFG-E8^−/−^ mice 2 months after BCAS. Scale bars: 20 μm. **B** Internalized dMBP volume per microglia in WT and MFG-E8^−/−^ BCAS mice (*n* = 3 for WT mice; *n* = 4 for MFG-E8^−/−^ mice; *P < 0.05). **C** Primary microglia were treated with rmMFG-E8 (200 ng/mL), cilengitide (5 nmol/L), and DID-stained myelin debris (100 μg/mL) for 2 h, primary microglia were collected, and the myelin debris signal intensity in microglia was measured by FACS (*n* = 4 repeats per group; **P < 0.01. **D**, E *Rac1* mRNA expression as tested by RT-PCR (**D**) and Rac1 activity measured by G-LISA (E) in primary microglia stimulated with PBS or rmMFG-E8 (500 ng/mL) for 3 h (*n* = 4 repeats per group for **D**; *n* = 3 repeats per group for E; * *P* < 0.05, unpaired *t*-test. All data are presented as the mean ± SD).

Myelin debris is an inhibiting factor for remyelination and needs to be removed for remyelination to occur. MFG-E8 is well-known as a bridging molecule to promotes the phagocytosis of apoptotic cells and amyloid-beta peptides by microglia in models of stroke and Alzheimer’s disease (AD). However, it is not clear whether MFG-E8 is able to improve the microglial phagocytosis of myelin debris. We detected the phagocytosis of myelin debris in the BCAS model by immunofluorescence. The dMBP wrapped by CD68 was defined as internalized dMBP. Compared to WT mice, MFG-E8^−/−^ mice exhibited a lower mean volume of internalized dMBP in CD68^+^Iba-1^+^ microglia, suggesting impaired phagocytic activity (Fig. 5A, B).

Extending our tissue results, we found a significant enhancement of myelin debris uptake after recombinant mouse MFG-E8 (rmMFG-E8) incubation with primary microglia *in vitro* (Fig. 5C). Previous studies have indicated that MFG-E8 binds to α_V_β_3_ and α_V_β_5_ integrins expressed on microglia. Then, Rac1, the downstream GTPase, is activated to regulate the actin cytoskeleton [21]. We also found that rmMFG-E8 enhanced the Rac1 activity without increasing its mRNA expression in primary microglia (Fig. 5D, E). To determine whether MFG-E8 mediates the engulfment of myelin debris through α_V_β_3_ and α_V_β_5_ receptors, a specific antagonist for α_V_β_3_ and α_V_β_5_ receptors, cilengitide, was added to microglia. Our results showed that the enhancement of phagocytosis by rmMFG-E8 was inhibited by cilengitide (Fig. 5C).

### MFG-E8-mediated Phagocytosis of Myelin Promotes Remyelination

**Fig. 6:**
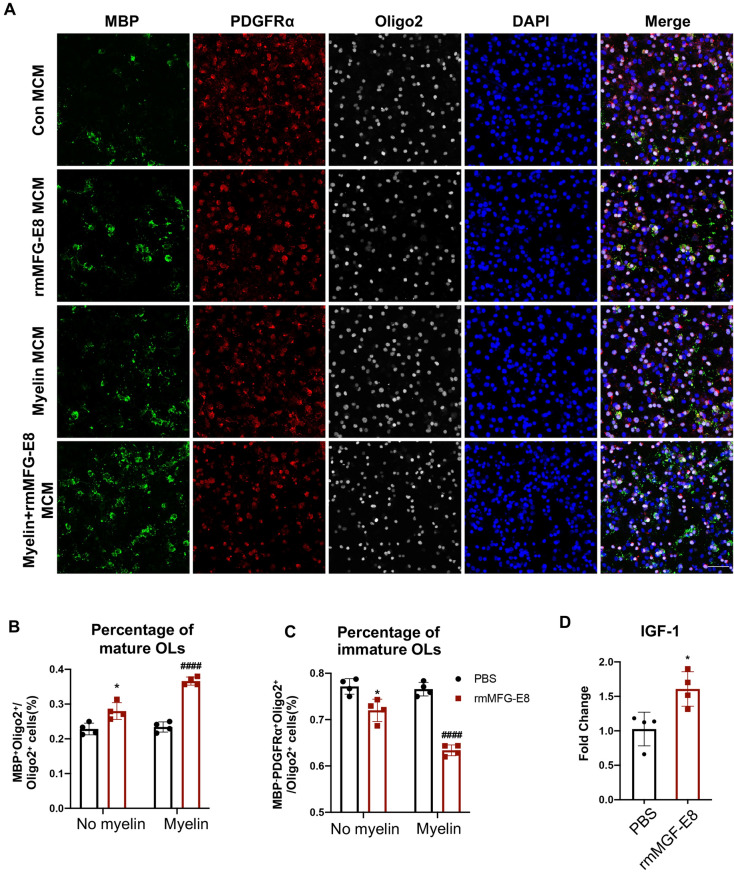
Recombinant mMFG-E8-treated microglia promote oligodendrocyte maturation *in vitro*. **A** Primary microglia were exposed to PBS, rmMFG-E8 (200 ng/mL), myelin debris (100 μg/mL), or rmMFG-E8+myelin debris for 6 h. Then the microglia-conditioned medium (MCM) was collected and added to OPCs with differentiating medium and incubated for 2 days. OPCs and OLs are labeled by immunofluorescence staining of MBP (green), PDGRFα (red), and Olig2 (grey). Nuclei are stained with DAPI. Scale bar: 50 μm. B, C The percentage of MBP^+^Oligo2^+^ OLs (**B**) and MBP^-^PDGFRα^+^Oligo2^+^ OPCs (**C**) of total Oligo2^+^ cells in PBS MCM, rmMFG-E8 MCM, myelin MCM, and myelin+rmMFG-E8 MCM treated OPCs (*n* = 4 per group; *P < 0.05 *vs* PBS MCM group, ^####^*P* < 0.0001 *vs* myelin MCM group. **D** IGF-1 mRNA expression in primary microglia treated with PBS or rmMFG-E8 (200 ng/mL) (*n* = 4 per group, unpaired *t*-test. All data are presented as the mean ± SD.

To determine whether MFG-E8-mediated myelin debris removal enhances the differentiation of OPCs into mature OLs, we applied myelin to stimulate microglia with or without rm-MFG-E8 incubation. Then the conditioned medium from microglia (MCM) was prepared and applied to the OPC cultures. The rmMFG-E8-treated MCM significantly elevated the proportion of MBP^+^ mature OLs and decreased the proportion of MBP^-^PDGFRα^+^Oligo2^+^ OPCs, with or without myelin stimulation (Fig. 6A–C). The conditioned medium of the rmMFG-E8 group, which did not contain myelin, also increased the proportion of mature OLs. This interesting phenomenon prompted us to conclude that the pro-differentiation effect of MCM on OPCs does not depend on the phagocytosis of myelin. Next, we found that MFG-E8 directly promoted the microglial expression of insulin-like growth factor-1 (IGF-1), a nutrient factor that promotes the differentiation of OPCs, suggesting a new mechanism by which MFG-E8 influences OL maturation (Fig. 6D). Otherwise, our results confirmed that MFG-E8 promotes the differentiation of OPCs *in vitro*.

### Astrocytes are the Main Source of MFG-E8

**Fig. 7:**
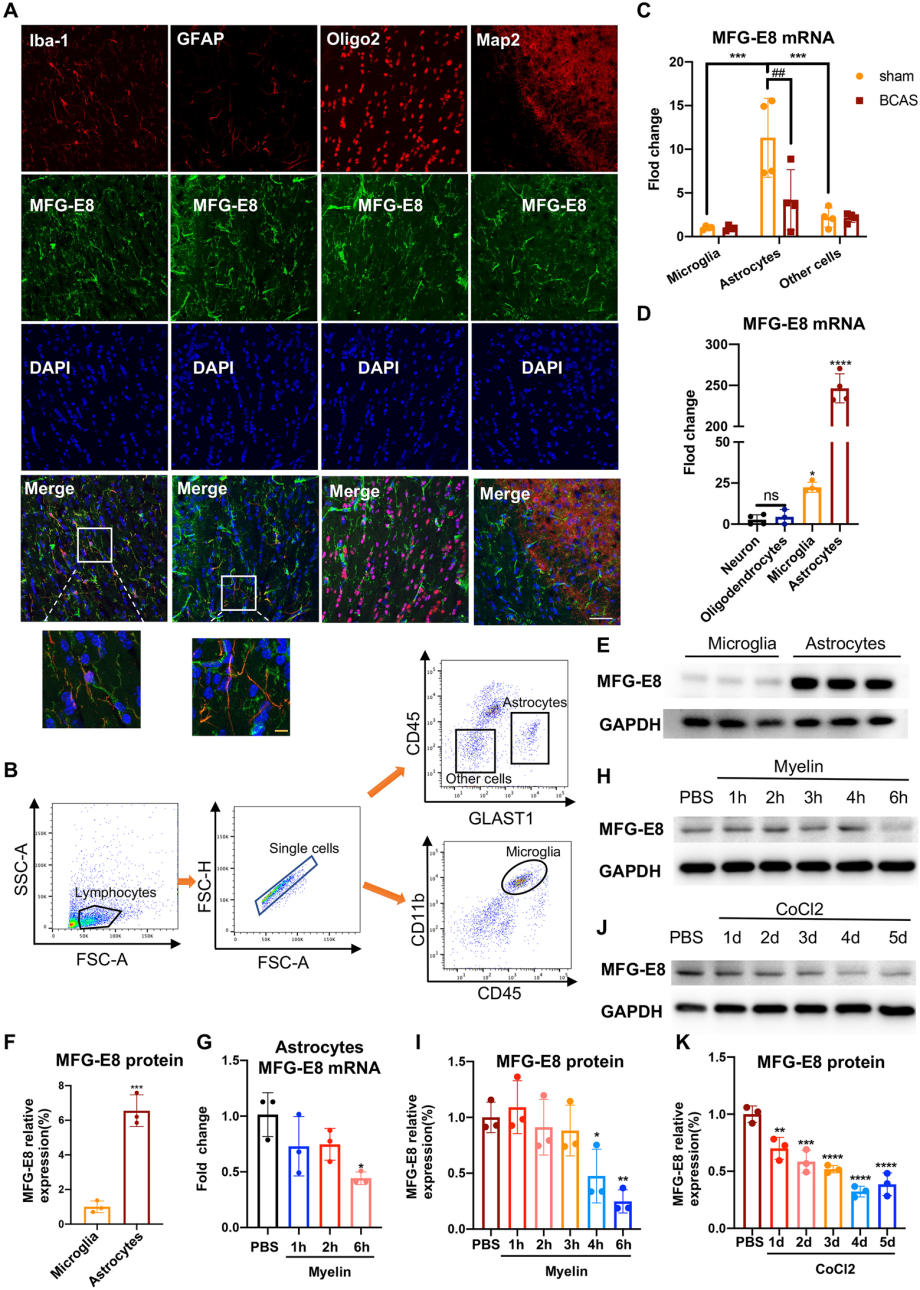
MFG-E8 is mainly expressed and secreted by astrocytes in the central neuronal system. **A** Representative immunofluorescence co-staining images of MFG-E8/Iba-1, MFG-E8/GFAP, MFG-E8/Oligo2, and MFG-E8/Map2 in the CC of WT sham mice. Microglia, astrocytes, oligodendrocytes, and neurons are labeled with Iba-1, GFAP, Oligo2, and Map2, respectively. White scale bar: 50 μm; orange scale bar: 10 μm. **B** Gating strategy for flow cytometry and FACS-sorting. **C** The mRNA levels of MFG-E8 in the microglia, astrocytes, and other cells from WT mice in the Sham and BCAS groups by qPCR (*n* = 4 per group; ***P < 0.001 *vs* astrocytes in sham group, ^##^*P* < 0.01 *vs* astrocytes in BCAS group, two-way ANOVA). **D** MFG-E8 mRNA expression in primary neurons, oligodendrocytes, microglia, and astrocytes without any treatment (*n* = 4 repeats per group; *P < 0.05, ****P < 0.0001 *vs* neurons, ANOVA). **E**, **F** Immunoblot bands (**E**) and quantification (**F**) of MFG-E8 expression in primary microglia and astrocytes with GAPDH as a loading control (*n* = 3 repeats per group; ***P < 0.001 *vs* microglia, unpaired *t*-test). **G** MFG-E8 mRNA expression in primary astrocytes at different time points after myelin stimulation (*n* = 3 per repeats group; *P < 0.05 *vs* PBS group, ANOVA). **H**, **I** Representative immunoblots (**H**), and densitometry analyses (**I**) of MFG-E8 expression in primary astrocytes at different time points after myelin stimulation (*n* = 3 per repeats per group; *P < 0.05, **P < 0.01 *vs* PBS group, ANOVA). **J**, **K** Representative immunoblots (**J**), and densitometry analyses (**K**) of MFG-E8 expression in primary astrocytes at different time points after CoCl2 stimulation (*n* = 3 repeats per group; **P <0.01, ***P < 0.001, ****P < 0.0001 *vs* PBS group, ANOVA. All data are presented as the mean ± SD).

Given the protective role of MFG-E8 in chronic cerebral ischemic injury, exploring its origin and increasing its secretion is important. Previous studies have demonstrated that MFG-E8 is expressed by a wide variety of cells, including macrophages, microglia, astrocytes, and neural stem cells [22]. Using immunofluorescence staining, we verified that MFG-E8 was expressed on Iba-1^+^ microglia and GFAP^+^ astrocytes, while there was no expression in Oligo2^+^ OLs and MAP2^+^ neurons (Fig. 7A). To investigate the predominant glial cell types expressing MFG-E8, we used flow cytometry to sort distinct populations from WT sham and BCAS mice after 2 months, including CD11b^+^CD45^median^ microglia, CD45^-^GLAST-1^+^ astrocytes, and CD45^-^GLAST-1^-^ other cells. Our analysis revealed that the expression of MFG-E8 mRNA was significantly higher in astrocytes than in microglia and other cells (Fig. 7B, C). Then, we examined the expression of MFG-E8 in primary neurons, OLs, astrocytes, and microglia *in vitro*. The mRNA expression of MFG-E8 in microglia and astrocytes was significantly higher than in neurons and OLs and was most expressed in astrocytes. Besides, the protein expression level of MFG-E8 in astrocytes was significantly higher than that in microglia (Fig. 7D–F). Our results showed that astrocytes may be the primary source of MFG-E8, rather than microglia, which is consistent with online human brain single-cell sequencing results (Fig. S1) from CELL TYPE EXPRESSION CORRELATES BASES (http://oldhamlab.ctec.ucsf.edu/).

Our *in vivo* experiments indicated that the decrease in MFG-E8 expression at 2 months after BCAS was mainly attributable to reduced expression in astrocytes (Fig. 7C). To test whether MFG-E8 expression in astrocytes could be decreased after myelin debris stimulation and chronic hypoxia, myelin debris and CoCl2 were used to stimulate the primary astrocytes. We found that the MFG-E8 mRNA significantly decreased 6 h after myelin exposure (Fig. 7G), and the protein expression of MFG-E8 was also down-regulated at 4 and 6 h after myelin administration (Fig. 7H, I). Similarly, CoCl2 stimulation significantly decreased the expression of MFG-E8 protein over time (Fig. 7J, K).

Taken together, our data demonstrated that MFG-E8 was mainly expressed and secreted by astrocytes and that chronic hypoxia inhibited its expression. Therefore, exogenous MFG-E8 administration has a certain application value.

## Discussion

WMI caused by hypoperfusion participates in the development of cognitive impairment or even dementia, resulting in a major social and economic burden. However, to date, there is a lack of effective treatments. We first found that MFG-E8 improved cognitive dysfunction after chronic cerebral ischemia by repairing WMI. At a mechanical level, MFG-E8 promoted remyelination through increasing myelin debris clearance and IGF-1 expression by microglia, which suggested that MFG-E8 is a potential therapeutic agent for chronic cerebral ischemic diseases-induced cognitive dysfunction.

We used the BCAS model to induce chronic cerebral hypoperfusion in mice. It has been reported that BCAS in mice has a significant reduction in cerebral blood flow, WMI in MRI, and a selective deficit in working memory [23, 24], which is consistent with our present findings. Besides, our previous study confirmed the positive correlation between the extent of working memory impairment and myelin loss in the corpus callosum of BCAS mice [25], suggesting that slowing or reversing WMI might be a promising therapeutic strategy for WMI-related cognitive impairments.

Myelin is mainly distributed in white matter and is vulnerable to ischemic damage. Remyelination is a self-repair process after demyelination, which restores electrical impulse conduction and metabolic and trophic support to axons [26]. In the chronic cerebral ischemia model, OPCs can respond immediately to myelin damage through proliferation and recruitment to the damaged area, and then differentiate to myelinating OLs to restore myelin. Accumulation of myelin debris is an important inhibitory factor for remyelination. Efficient myelin debris clearance by phagocytic cells is critical to OPC differentiation into myelinating mature OLs [27]. Microglia are mainly phagocytes in the CNS. Several receptors expressed by microglia that promote the phagocytosis of myelin debris have been identified, including fractalkine receptor CX3CR1, Triggering Receptor Expressed on Myeloid cells 2, Toll-Like Receptor 4, Retinoid X Receptor-α, and tyrosine-protein kinase MER [28–33]. Our results identified MFG-E8 as a promoting factor for remyelination by enhancing the phagocytosis of myelin debris by microglia both *in vivo* and *in vitro*.

MFG-E8 is a bridging protein and has two epidermal growth factor (EGF)-like domains in the mouse that recognize the integrins α_V_β_3_ and α_V_β_5_ mainly expressed on phagocytes and two discoidin domains that recognize phosphatidylserine (PS) exposed on the cell membranes of apoptotic cells [22, 34]. The unique structure of MFG-E8 enables it to act as a tether to attach the debris and apoptotic cells to the phagocytes. In stroke, Aβ stimulation and *Brucella abortus* infection models, MFG-E8 mediates microglial the phagocytosis of PS-turnover neurons whether they are viable or dead [14, 35–37]. Myelin is rich in lipids containing PS. We first found, using flow cytometry, that MFG-E8 promoted the phagocytosis of myelin debris by microglia, which is the probable mechanism of myelin regeneration. MFG-E8 is largely expressed in microglia, while a small amount is expressed in neurons and astrocytes [15, 38]. Our *in vivo* and *in vitro* experiments confirmed the expression of MFG-E8 in microglia and astrocytes. However, the MFG-E8 in neurons was undetectable, which may be a result of the stained sites. MAP2^+^ axons in the corpus callosum may not express MFG-E8, whereas NeuN^+^ neuronal cell bodies in the cortex do. However, the expression of MFG-E8 in astrocytes is significantly higher than that in microglia. MFG-E8 may be mainly derived from astrocytes. The human brain single-cell sequencing data from public databases also supports our findings. We suspect that MFG-E8 is a communication molecule between astrocytes and microglia, and MFG-E8 secreted by astrocytes binds to microglial α_V_β_3_ and α_V_β_5_ receptors, thereby enhancing the ability of microglia to engulf myelin fragments and promoting white matter repair.

The integrins α_V_β_3_ and α_V_β_5_ are acknowledged to be MFG-E8 receptors. The EGF-like domain of MFG-E8 contains an RGD motif that binds the α_V_β_3_ and α_V_β_5_ integrins. Integrin activation is associated with signaling to Rho-GTPases, Rac, and Cdc42 which are involved in the regulation of the actin cytoskeleton [39]. In dendritic cells, MFG-E8 binds to the α_V_β_5_ integrin, which induces the recruitment of the CrkII-DOCK180-Rac1 complex and thus activation of Rac1. Rac1 activation results in cytoskeletal reorganization and plays an important role in cell migration and phagocytic activity [21]. In our study, the enhancement of microglial clearance of myelin debris and Rac1 activity by rmMFG-E8 was diminished after blocking the α_V_β_3_ and α_V_β_5_ receptors by cilengitide, which is consistent with previous studies.

Previous studies have shown that medin aggregates (a fragment of MFG-E8) are deposited in the aged blood vessel wall, thereby increasing vascular amyloid deposition, participating in the pathogenesis of cerebral amyloid angiopathy and AD, and promoting the development of cognitive impairment [40–42], which is inconsistent with the findings of our study showing that MFG-E8 can improve BCAS-induced cognitive impairment. There may be several reasons for this. First, Medin is not equivalent to MFG-E8. It is a 50 amino-acid amyloid derived from the C2 domain of the MFG-E8 protein. It is believed that medin can be deposited in blood vessels, induce vascular inflammation, promote Aβ deposition, and thus participate in the pathogenesis of vascular dementia and AD, thus undertaking a biological function different from MFG-E8. Second, medin is not a domain by which MFG-E8 plays a phagocytotic role. A previous report found that knocking out the MFG-E8 C2 domain reduces the deposition of medin in the vasculature, but it does not reduce its expression in astrocytes, nor does it affect the phagocytic function and inflammatory factor release action of microglia [41]. Therefore, we believe that the phagocytosis of myelin debris and the cognitive protection by MFG-E8 after BCAS are independent of the medin protein. Further research is needed to explore the mechanism by which medin is cleaved from MFG-E8 and avoids MFG-E8 becoming an accomplice of Aβ. Our study provides new insights into the potential therapeutic role of MFG-E8 in cognitive impairment, and we look forward to further investigations in this area.

We first found that MFG-E8 directly increased the expression of IGF-1 in microglia, which is probably another mechanism by which MFG-E8 promotes remyelination. IGF-1 promotes OL development and myelin production [43]. Transgenic mice continuously expressing IGF-1 exhibit more rapid remyelination than control mice after cuprizone-induced demyelination resulted from an increased survival of mature OLs [44]. The results of our experiments indicate that, besides promoting microglial phagocytic activity, the MFG-E8-induced production of IGF-1 by microglia may also support myelin regeneration. However, the mechanisms by which MFG-E8 up-regulates IGF-1 expression in microglia are not clear yet and require further study.

In addition, MFG-E8 has some characteristics showing potential for clinical transformation. First, it is produced and secreted by cells. In systemic lupus erythematosus patients, especially with cerebrovascular disease, serum MFG-E8 is elevated [45]. Moreover, it could be tested in the cerebrospinal fluid (CSF) of patients with frontotemporal lobar degeneration (FTLD), indicating a potential CSF biomarker to discriminate FLTD and its pathological subtypes [46]. The endogenous property of MFG-E8 avoids immunological rejection after administration. Second, an exogenous rhMFG-E8 supply has been applied in mice stroke models and plays a protective role in ischemic injury after transvenous injection. In addition, an injectable MFG-E8-loaded copolymer system has been reported and applied to mice with spinal cord injury, where it had a neuroprotective effect [47].

There are some limitations to our study. First, in the *in vitro* study, microglia were exposed to MFG-E8, and different concentrations of MFG-E8 need to be further applied to explore the optimal dose. Second, MFG-E8 has been reported to be involved in microglial polarization and the inflammatory response [48, 49]. Although microglial phagocytosis of myelin debris or apoptotic cells can affect its phenotype and polarization, our study only focused on the effect of MFG-E8 on microglial phagocytosis and remyelination; the inflammatory response mediated by microglia and whether it is related to the phagocytosis of myelin debris by MFG-E8 still needs to be further studied.

## Conclusions

In summary, the current data demonstrated that MFG-E8 alleviated WMI-related cognitive dysfunction by facilitating remyelination in mouse BCAS models. A possible mechanism for remyelination is that MFG-E8 promotes the microglial phagocytosis of myelin debris and IGF-1 production in microglia. Therefore, we suggest that MFG-E8 with its endogenous and exocrine properties serves as an encouraging therapy for cognitive impairment after chronic cerebral hypoperfusion.

### Supplementary Information

Below is the link to the electronic supplementary material.

Supplementary file1 (PDF 152 kb)

## Data Availability

All data are available upon request to the corresponding authors.
