# Supplementary material for: MFG-E8 Alleviates Cognitive Impairments Induced by Chronic Cerebral Hypoperfusion by Phagocytosing Myelin Debris and Promoting Remyelination
Source: Neurosci Bull. 2023 Nov 18;40(4):483–99. doi: 10.1007/s12264-023-01147-1 (PMC11003935; doi:10.1007/s12264-023-01147-1)
Supplement: Supplementary file 1 — Supplementary file1 (PDF 152 kb) [file 12264_2023_1147_MOESM1_ESM.pdf]

Supplementary Information

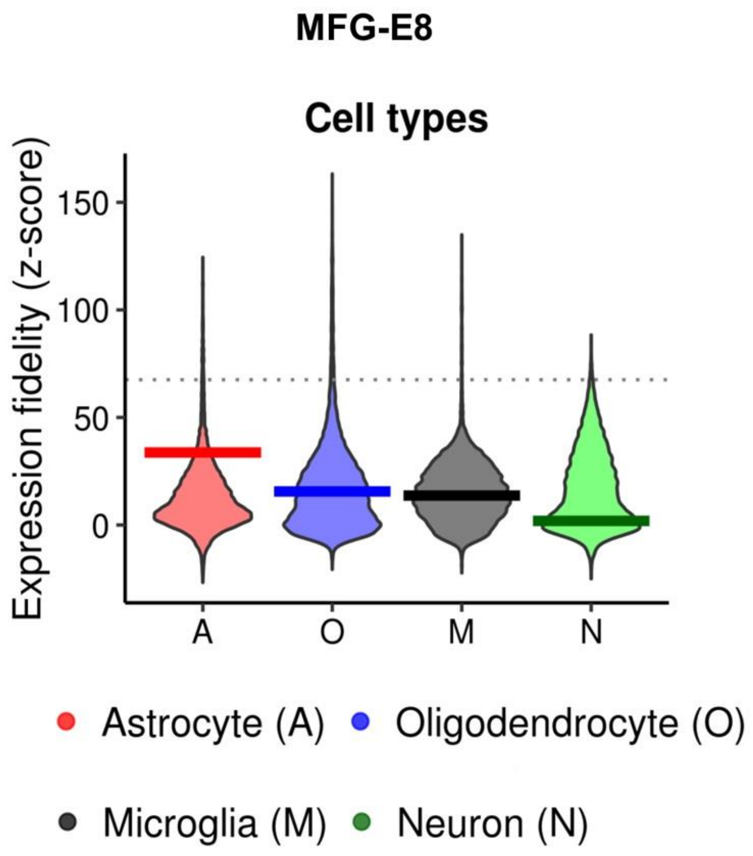

Fig. S1 Human brain single-cell sequencing results from CELL TYPE EXPRESSION CORRELATES BASES. Genome-wide distributions of expression fidelity for astrocytes (A), oligodendrocytes (O), microglia (M), and neurons (N) over all analyzed samples are shown. The horizontal line denotes the expression fidelity of the query gene for each cell type. The dashed horizontal line denotes the threshold above which all fidelity scores had Confidence = 100.
